# Emotional reaction to diagnosis of infertility in Kuwait and successful clients' perception of nurses' role during treatment

**DOI:** 10.1186/1472-6955-9-5

**Published:** 2010-03-18

**Authors:** Florence E Omu, Alexander E Omu

**Affiliations:** 1 College of Nursing, The Public Authority for Applied Education and Training, Shuwaikh, PO Box 23167 Safat, 13092, Kuwait; 2 Department of Obstetrics & Gynecology, Faculty of Medicine, Health Sciences Center, Kuwait University, Jabriya, 4th Ring Road, PO Box 24923 Safat, 13110, Kuwait; 3 Maternity Hospital Kuwait, Ministry of Health, Al-Sabah health region, Abdel-Nasser Road, West Shuwaikh, PO Box 5, Suleibikat, 13001, Kuwait

## Abstract

**Background:**

The unfulfilled desire of millions of infertile couples worldwide to have their own biological children results in emotional distress. This study evaluated the emotional reactions of couples attending a combined infertility clinic in Kuwait and successful clients' perception of nurses.

**Methods:**

Quantitative and qualitative methods were used. The first phase was by structured interview using two standardized psychological scales: the 25-item Hopkins Symptom Checklist and Modified Fertility Adjustment Scale. Data were collected from 268 couples attending the combined infertility clinic, between October 2002 and September 2007. The second phase was a semi-structured interview of 10 clients who got pregnant following treatment. The interview explored their feelings and perception of the nurses' role. Interviews were transcribed verbatim and analyzed.

**Results:**

The average duration of infertility was 4 years; 65.7% of the women and 76.1% of men suffered from primary infertility. Emotional reactions experienced were: anxiety in women (12.7%) and men (6%), depression in women (5.2%) and men (14.9%) and reduced libido in women (6.7%) and men (29.9%). Also in men, 14.9% experienced premature ejaculation, 5.2% weak ejaculation and 7.9% had impotence although 4.9% were transient. In the semi-structured interviews, the emotions expressed were similar and in addition to anger, feelings of devastation, powerlessness, sense of failure and frustration. In the survey, 12.7% of the men were found to show more anxiety than women (6%). Although all the 10 women interviewed confirmed they were anxious; only 4 of their partners were reported to be sad or anxious. Successful clients' perception of nurses' roles included nurses carrying out basic nursing procedures, communicating, educating about investigative and treatment procedures, providing emotional support by listening, encouraging, reassuring and being empathetic.

**Conclusions:**

This study illuminates the emotional reactions of infertile clients. Fertility nurses in Kuwait can provide emotional support through communication. The need for additional and continuous training for nurses employed in fertility settings in Kuwait is paramount.

## Background

Statistics on the prevalence of infertility vary worldwide; the infertility rate in America was reported as 11.2% in 1965, however, in 2002, it was believed to have decreased to 7.4% [1]. A review of infertility rates reported in 25 population surveys from 1990-2006 from both developed and developing countries estimated 72.4-120.6 million women aged between 20-44 years, in heterosexual relationships and desiring to have their own biological children were infertile. The same review also estimated that 40-90.4 million of these women received infertility treatment [2]. Assisted Reproductive Technology (ART) is now a multi billion dollar industry [3].

Infertility management should be viewed holistically because the medical, psychological, spiritual and socio-cultural components of infertility are inseparable and need to be addressed simultaneously. The medical components of infertility are often emphasized at fertility treatment centers unlike the other components. Some clients have unexplained infertility and will benefit from psychological interventions. Psychological stress is inevitable for the infertile clients and there are different views about how it affects the outcome of treatment. Infertility specialist doctors have large caseloads, making extensive consultation time with each client difficult to arrange. Some clients, unsatisfied with limited consultation time perceive doctors as only being concerned with treating underlying medical or gynecological problems and not providing emotional support by 'consoling' them or addressing their emotional problems [4-6].

A 30 year systematic review of literature from 1966 found 1,957 articles and numerous books published on the psychological aspects of infertility. In 15 of these studies pregnancy was used as the indicator for successful outcome of infertility interventions [7]. The author compared studies of clients undergoing infertility treatment complemented with psychological interventions of either client education or counseling. The result showed that educational interventions were better than counseling interventions [7]. The Human Fertilization and Embryology Authority (HFEA) Code of Practice identifies three types of compulsory counseling for clients undergoing In-Vitro Fertilization (IVF): Implication counseling to educate them on the implications of their treatment option; support counseling to assist them during the period of emotional stress; and therapeutic counseling to help them cope with the consequences of treatment and adjust their expectations and acceptance of their situation [8]. This supports the rationale for suggesting that counseling for IVF clients should be provided by specialist doctors, because these women are seeking clarification of their problems more than just emotional support [9]. This may be true, but most infertile clients at different times during their treatment also need emotional and social support, which nurses can provide. The nature of nursing puts nurses in a position of providing continuous client care, unlike medical and other health professionals. The continuous nursing presence has been reported as "holding together multiple components of ART process, including the clients' emotional and physical experiences, the different roles of the different specialist team members and also the nurses' emotions" [10]. Emotional and educational support from nurses would complement all other interventions for infertile clients undergoing treatment. Nurses' educational interventions will reinforce the specialist doctors' educational interventions. In the United Kingdom, the Royal College of Nursing (RCN) has published guidelines for Nurses, for IVF counseling emphasizing the need for emotional support [11]. Regulatory bodies for nurses also strive to make nursing relevant by emphasizing the expanded nursing roles in different nursing specializations, including fertility nursing. Post registration studies in infertility nursing are being offered in many institutions in the United Kingdom covering aspects of infertility problems, nursing interventions and treatment [12].

The religious and cultural perspectives of infertility can be challenging for some clients. In Kuwait, the Islamic religion believes that most things come from Allah (God) and expresses acceptance of God's will by utterances like 'insha'allah' meaning God willing, 'al-hamdu li-llah' meaning thanks be to God [13]. An infertile couple might be consoled because Allah wishes for them to be childless. In Kuwait it is customary to identify adults by placing the prefix 'Abu' or 'Om' with their child's name, for example, Abu Khalid or Om Mohammed meaning Father of Khalid or mother of Mohammed respectively. This custom serves is a reminder to the infertile couple of their social obligations to procreate and results in personal frustration. Some studies discuss the effect of pressure from family members and the community affecting the quality of life of infertile couples [14]. Men and women are believed to react differently to infertility. Infertile wives are said to experience greater emotional disturbances than their husbands because, during the process of female socialization, pregnancy and childbirth are considered as the most important function of the wife. Females, therefore, take more responsibility for fertility evaluation even when they are sure that their husbands are the cause of their childlessness. Some of the investigations and treatment for artificial reproductive technology are performed on females, so they directly face the success or failure of treatment [15-18]. Men are said not to react in the same way as women because male socialization discourages them from expressing feelings openly. However, if there is evidence that the man is the cause of the reproductive impairment, he would be more distressed than his wife [5,19,20].

There is a large literature on infertility diagnosis and interventions, but there are fewer studies on the role of nurses during infertility management. Some of the studies include quantitative surveys on the role, or expanded roles and responsibilities of nurses working in infertility settings [21,22], qualitative ethnographic studies on the role of the nurse during fertility treatment [4] and qualitative interpretative study interviewing infertility nurses regarding their roles during infertility management [10]. We did not find studies reporting clients' perception of nurses' role during their infertility treatment.

The aims of the study are twofold: first to evaluate the emotional reactions of couples attending a combined infertility clinic in Kuwait; second to fill the knowledge gap by examining the perception of successfully treated clients regarding nurses' roles during their treatment.

## Methods

Two types of interviews were used for data collection: firstly, a quantitative survey method using two structured standardized psychological interview scales: the 25-item Hopkins Symptom Checklist (HSCL-25) and Modified Fertility Adjustment Scale (MFAS) [23,24]. The internal consistency of HSCL-25 and MFAS were 0.81 and 0.8 respectively. The second type of interview was a semi-structured interview of clients who got pregnant after infertility treatment. This was to explore their feelings towards their infertility diagnosis and their perception of nurses' role during their infertility treatment period.

The Setting of the study was at the Maternity hospital Kuwait. It is 400-bedded government hospital providing gynecological, obstetrics, specialist infertility clinics, an IVF unit and neonatal care unit [25]. Nurses working in these clinics and IVF unit are registered staff nurses/midwives with no special training for infertility problems, counseling or nursing interventions for infertile couples. Their roles are best described as care-giving, clerical and receptionist, assisting doctors with physical examinations, translation and collection of specimens.

### Ethical considerations

Approval for the study was obtained from the ethical committee of the hospital before starting the study. All participants were told that participation was voluntary and non-participation will not affect their treatment. Only couples volunteering were interviewed.

The sample in the first phase consisted of 268 married couples recruited from the combined infertility clinic, Maternity Hospital between October 2002 and September 2007. The sample size required for adequate power (80%) to detect significant differences (0.8 standard deviation) in family stress outcomes of respondent groups with significance level of 5% was calculated to be approximately 250 respondents [26]. The purpose of the study was explained to all consented couples. Couples were interviewed about their demographic characteristics and the effects of infertility on their sexual performance. The concerns of their relatives and the emotional reaction of the clients to infertility in terms of anxiety and depression were evaluated.

In the second phase, 10 women who became pregnant following their infertility treatment were invited to share their experiences and perception of nurses' roles during their treatment. Survey instrument was used in phase 1, couples attending the specialized clinic were interviewed together to ensure accuracy of their responses; although, different checklists were completed for husband and wife by the consultant doctor (one of the researchers). HSCL-25 is a valid and reliable checklist used to screen for emotional distress (symptoms of anxiety and depression) in non-psychiatric hospital outpatients. The choice of the HSCL-25 was predicated on previous experience with the HSCL-25, which has been successfully used in previous studies in Kuwait among prenatal clients [27]. The reliability and validity of HSCL-25 have been tested and proven to be useful among displaced persons in several countries [28-31]. The estimated Cronbach's a for depression was 0.85 and 0.86 for anxiety. The study protocol included demographic characteristics, medical and gynecological histories, age, duration and type of infertility. The results of investigations carried out for the evaluation of the infertility were extracted from their medical files and initial interviews. In using the HSCL-25, scores were computed by averaging the degree of distress reported for anxiety and depression symptoms (none = 0; mild = 1, moderate = 2 and severe = 3). Score above 1.50 were accepted as clinically significant levels of anxiety and depression. The evaluation of the sexual performance of the couple was carried out using MFAS [24]. The prevalence of impaired libido, premature ejaculation and transient or occasional impotence where elicited. The scale was scored from, 0 = none, little = 1, some = 2, moderate = 3, very = 4 and extreme = 5, with a score of MFAS of 3 or higher were considered to reflect significant family stress.

The second phase was the qualitative interview involving 11 clients (9 women and 1 couple) representing 10 successful ART couples. Pilot testing of the interview guide for suitability of the questions was by 2 women who had undergone ART treatment (one successful and the other failed ART). The 10 female participants with successful ART treatment were contacted by telephone and, since they were part of the initial study, they agreed to share their experiences with the female researcher. The participants chose the venue for interview. Three preferred to be interviewed at home while 7 of them preferred to be interviewed in a quiet office at their workplace. Interviews lasted 35-45 minutes and were tape recorded. The questions were asked using semi structured format. The interview schedule is in the appendix. Further questions as needed were asked allowing participants to provide as much information as they were willing to disclose. Sufficient time was given for subjects to elaborate. A digital voice editor was used to transcribe verbatim the interviews. Participants were given the transcript of their interview to correct any misunderstanding and add any omissions. They all confirmed that the transcripts covered their main perceptions. Data were analyzed by both researchers using 6 phases of thematic analyses: familiarization with the data, generation of initial codes, searching for themes, reviewing themes, defining and naming themes and producing the report [32].

Quantitative data were analyzed using the Statistical Package for Social Sciences (SPSS). Frequencies, standard deviations, chi-square test and Fisher Exact (non-parametric) tests were used to examine the relationship between the variables.

## Results

### Demographic characteristics of the main Sample

The majority of the women, 249 (92.9%) were between <19-39 years old (Table 1). The men were significantly older than the women with mean age of 34.6 ± 6.3 versus 28.9 ± 4.1 for women (p < 0.05). In both men and women the average duration of infertility was approximately 4 years although slightly higher in men. Similarly, primary infertility occurred in 176 (65.7%) of the women and 204 (76.1%) of the men compared with 92 (34.3%) and 64 (23.9%) for secondary infertility. Understandably, indigenous Kuwaitis made up the majority of the study subject for both genders. All respondents had some form of formal education, with 91% of men and 92% of women respectively having secondary school education and about 25-30% of both couples having university education. Significantly more men were smokers than women (44% versus 8%, p < 0.001).

**Table 1:** Characteristics of the survey participants

Characteristics	Women N = 268	%	Men N = 268	%	P value
**Age in years:**					

**<19**	4	2	0	0	0.124*

**20-29**	126	47	5	1.90	0.001*

**30-39**	123	45	118	44	0.728

**40-49**	15	5.6	126	47	0.001*

**>50**	0	0	19	7.10	0.001*

Ethnic Groups					

Kuwaitis	182	67.9	178	66.4	0.783

Asians	42	15.7	40	14.9	0.905

Other Arabs	39	14.60	48	17.9	0.349*

Others	5	1.9	2	0.80	0.045*

Education:					

Primary School	21	7.8	24	8.9	0.755

Secondary School	162	60.50	176	65.7	0.245*

University/College	85	31.7	68	25.4	0.126*

Smoking:					

Heavy smoker: >20 cigarettes/day	15	5.6	52	19.4	0.001*

Light smoker <20 cigarettes/day	6	2.20	65	24.3	0.001*

Duration of Infertility in years:					

<1	2	0.80	2	0.80	1.376

1-2	158	59	144	53.7	0.258*

3-4	48	17.9	52	19.4	.739

5-6	27	10.10	33	12.3	0.493*

7-8	23	8.6	23	8.6	1

>8	10	3.7	14	5.2	0.532

Secondary	92	32.3	64	23.9	0.010*?

* = p < 0.5

The main causes of infertility in both men and women are shown in Table 2. Among the men, azoospermia was significantly associated with reduced libido (p < 0.01) and depression (p < 0.01).

**Table 2:** Association between causes of infertility and emotional reactions

Causes of Infertility		No of client	%	Anxiety	%	Depression	%	P value
Women		N = 268		34	12.7	4	5.2	0.001*

Ovarian dysfunction		174	64.9	24	13.8	7	4.9	0.002*

Tubal blockage		16	6	2	12.5	1	4	1

Endometriosis		15	5.6	1	6.7	2	13.3	1

Uterine factor		6	2.2	0	0	1	1.6	1

No female factor		57	21.3	7	12.3	3	5.3	0.321*

Men		N = 268		16	6	40	14.9	0.001*

Sperm Count:								

Normozoospermia		164	61.2	8	4.9	17	10.4	0.045*

Oligozoospermia		80	29.9	6	7.5	8	10	0.780

Azoospermia		24	8.9	2	8.3	15	62.5	0.002*

Motility:								

Normal		188	70.2	11	5.8	29	15.4	0.004*

Asthenozoospermia		80	29.8	5	6.3	11	13.8	0.186*

Morphology:								

Normal morphology		237	88.4	13	5.5	32	13.5	0.005*

Teratozoospermia		31	11.6	3	9.7	8	25.8	0.182*

Immunological:								

No antisperm antibodies		220	82.1	14	6.4	34	14.6	0.004*

Antisperm antibodies		48	17	2	4.2	8	16.7	0.091*

* = p < 0.5

Heavy smoking (i.e. more than 20 cigarettes per day) was significantly associated with anxiety (p < 0.01). Infertility of 1-2 years was significantly associated with anxiety among both the men (p < 0.05) and the women (p < 0.01) and premature ejaculation in the men (p < 0.05).

In Table 3, the association between psychosexual problems and emotional reactions of couples to infertility was analyzed. Indicators identified for the analysis were anxiety, depression, reduced libido and premature ejaculation. Women were more prone to anxiety than men (p < 0.01). Conversely, the men tended to have more depression (p < 0.01). There was significant association between duration of infertility of five years and more with loss of libido (p < 0.01) and depression among the men (p < 0.05).

**Table 3:** Association between psychosexual problems and emotional reactions of both men and women to infertility

	Women	Men	P value		
**Emotional/psychosexual problems**	**N = 268**	**%**	**N = 268**	**%**	

Anxiety	34	12.7	16	6.0	0.012*

Depression	14	5.2	40	14.9	0.001*

Anxiety + Depression	10	3.7	9	3.4	1

Reduced libido	18	6.7	80	29.9	0.001*

Anxiety + Reduced libido	5	1.9	11	4.1	0.001*

Depression + Reduced libido	4	1.5	9	3.4	0.261*

Premature ejaculation	-	-	40	14.9	-

Weak/impaired ejaculation	-	-	14	5.2	-

Impotence:	-	-	21	7.9	-

Transient	-	-	13	4.9	-

Persistent	-	-	8	3.0	-

Menstrual Problems:	77	28.7	-	-	-

Oligomenorrhea	48	17.9	-	-	-

Menorrhagia	17	6.3	-	-	-

Metrorrhagia	12	4.5	-	-	-

* = p < 0.5

The most common problem was reduced libido in 29.9% of the men and 6.7% of women (p < 0.01). However, of the 80 men initially volunteering information about reduced libido, only 17% attributed it to the problem of infertility. Although more women than men had anxiety reaction, more men than women had anxiety plus reduced libido and depression plus reduced libido (p < 0.05). Premature ejaculation occurred in 14.9% of the men ranging from 6 months to 5 years but only 15% attributed it to their infertility. Impotence was found in 7.9% of the men. However, more than half the men (4.9%) had transient or occasional impotence only, while impotence was persistent in 8 men (3.0%). Weak/impaired ejaculation occurred in 5.2% of men without a previous history of diabetes mellitus. In five men, there was non-consummation of the marriage of between 2 to 5 years. Approximately 29% of the women had severe menstrual problems in form of Oligomenorrhea, Menorrhagia and Metrorrhagia. There was a strong association between menstrual disorders; Oligomenorrhea and anxiety (p < 0.01), and between Menorrhagia and depression (p < 0.05).

### Result of the semi-structured interview of clients who got pregnant after infertility treatment

Participant profiles are provided in Table 4

**Table 4:** Profile of qualitative participants

#	Pseudonyms	Sex	Age in years	Duration of Infertility in years	Ethnic group	Religion	Occupation	Type of infertility and special remark
1	Ahmed	M	40	5	Others	Moslem	Engineer	Couple with primary infertility

2	Muneera	F	33	5	Others	Moslem	Nurse	Couple with primary infertility

3	Rachael	F	32	3	Asian	Christian	Teacher	Primary infertility

4	Sara	F	35	8 1/2	Other Arab	Christian	Librarian	Secondary infertility

5	Kitty	F	38	7	Others	Christian	Pharmacists	Primary infertility

6	Zaina	F	39	5 1/2	Kuwaiti	Moslem	House wife	Primary infertility

7	Nancy	F	27	1	Asian	Christian	Microbiologists	Primary infertility

8	Pamela	F	36	3	Asian	Hindu	House wife	Secondary infertility

9	Abeer	F	28	3	Kuwaiti	Moslem	House wife	Primary infertility

10	Mary	F	37	12	Others	Christian	Female priest	Primary infertility

11	Alia	F	32	2	Kuwaiti	Moslem	Lab technician	Primary infertility

The major topics selected were those mostly emphasized by most of the participants. Although many topics emerged from the qualitative interviews; only those relevant to the 2 aims of the study: emotional reactions to infertility and clients' perception of nurses' roles during fertility treatment were analyzed.

### Emotional reactions before and during infertility treatment

The 4 sub topics that emerged included: the female clients' reactions, their husbands'/partners' reactions, their family reactions and their coping strategies before they became pregnant.

The reactions of the women before they became pregnant included anxiety, depression, anger, feeling devastated, powerlessness, sense of failure and different degrees of frustration including overwhelming frustration. Below are some quotes expressing their frustrations before they became pregnant:

*'It wasn't a nice feeling at all, each month when I was expecting to get pregnant and I start menstruating, I have "big headache", wondering why it should come and for how long I will have to cope with pressure from our families'*.

(Muneera, 33 yrs old with 5 years of primary infertility)

*'When I crossed the 5 years period I set for myself, my patience ran out. I remember one day when my period came, I was very frustrated and fully upset, I went to consult my doctor and he tried to console me by asking me to be patient because all my test results were normal. I couldn't take it any longer, oops !!!, I was high that day and I lost it, I told him not to talk to me about patience, I knew all about patience and I can define patience better than the Oxford dictionary, I was overwhelmed with frustration, I went home and just cried uncontrollably" *(Mary, 37 years old with 12 years of primary infertility).

In both quotations above, participants mentioned their frustration at the onset of their menstruation, which was an indication that they were not pregnant. Both participants were above 30 years with more than 5 years of primary infertility. Although the younger participants less than 30 years old or with fewer than 3 years of infertility did not express such devastation, they were also frustrated. Below is a quotation from a 27 year old participant and another from a participant with fewer than 3 years of infertility:

*'I was very upset and frustrated, really, I knew it was my problem and I wanted to get help quickly'*.

(Nancy, 27 years old, with 1 year of primary infertility)

*'I was anxious but I was aware of my problems and wanted it fixed as soon as possible'*.

(Alia, 32 years old with 2 years of primary infertility).

*'I felt depressed and had a sense of failure. My husband was partly the cause of our fertility failure, but he appeared to be ok, not looking stressed'*.

(Kitty, 38 years old with 7 years primary infertility).

It would appear that when reproductive failure is as a result of combined male and female factors, the woman is also affected emotionally even when her spouse appears not bothered.

Some participants with female factor infertility but with very supportive spouses coped relatively well, like in the case of Rachael:

*'The time I felt useless. Knowing that I cannot give child to my husband made me feel so bad, and I thought that something was missing on my part'*. *'My husband was ok, and he said he will not leave me if I cannot give him a baby'*.

(Rachael, 32 years with 3 years primary infertility).

The woman's age appears to be a factor in their emotional reaction because they were all aware of their biological clock and were in a hurry to get a baby before it became too late. The other factor that might have been of concern to some of the women is the fear of losing their spouses to another woman who may be able to prove their fertility by getting pregnant.

Two of the participants had secondary infertility, but the condition of their child affected the couples' reactions. Whereas, Pamela, 36 years old with 3 years secondary infertility and a normal child did not appear to be under great pressure before she got pregnant. Sara, 35 years old with 8 1/2 years of secondary infertility and a brain damaged child, appeared to be extremely frustrated before she finally got pregnant. She said:

*'I felt depressed and devastated because my only son had brain damage at birth, he is now 8 1/2 years'*. When asked about her husband's reaction she said: '*After the 5*^*th*^*failed IVF, my husband suffered a heart attack because of the tension, now he suffers from hypertension and diabetes'*.

### Husbands'/partners' and family reactions

The only male participant said:

*'I felt sad, because generally speaking, when couples are married, until they have children, my culture does not consider their marriage successful. This is bad because it is God that gives children and not the couple and the basis of marriage is supposed to be love'*. (Ahmed)

*Alia said: 'My husband was very upset and embarrassed especially when some- one accused him of being lazy and not able to get me pregnant, he then insisted that we seek medical help'*.

The above quotations illustrate the amount of pressure that is sometimes endured by infertile men, whether or not the reproductive failure was entirely their fault or that of their wives.

Family reactions also varied and in some cases caused added frustration.

*'The expectations of our parents to have grand children were very high and this led to constant pressure on us' *(Ahmed & Muneera).

*'His family was not worried and they were not pressurizing us, but they were suggesting many things including special prayers. My parents were concerned and after 9 years my mother arranged for me to adopt a child'*. (Mary).

### Their coping strategies before they became pregnant

Nine of the female participants said that they could not cope psychologically until they became pregnant. Mary with 12 years of primary infertility said: *'during the first 5 years, It was my faith in God and my pastoral work (praying and empowering other people with problems) that kept me going. After the 9*^*th*^*year, it was our adopted son that really calmed us and our lives revolved around him'*.

### Clients' perception of nurses' roles during their infertility treatment

The second major theme was clients' perception of nurses' roles during their fertility treatment.

The generally expressed view was that nurses have a role in infertility treatment. They all mentioned the following roles: monitoring their vital signs and weight, administering oral medications and injections, being with the doctors during consultations and assisting in physical examinations, consoling and encouraging them. The term 'nurses encouraged them', had different connotations including: reassuring, communicating, counseling and educating them. Nurses were also said to prepare them for investigations; some mentioned collecting samples. Specifically with clients who attended the IVF units, they reported that the nurses, in addition to the roles already mentioned, performed additional roles which included: clarifying their doctors' instructions regarding their treatment options, assisting the doctor during embryo transfer and staying with them for one hour following embryo transfer. During this period they said the nurses provided them with health teachings regarding the restrictions they must observe to ensure successful treatment. The following were quotes from some of the clients:

*'This particular nurse was always very supportive, she helps me onto the couch, she holds my hand during the procedures and makes me relax during some rather embarrassing procedures'*. (Kitty)

*'Sometimes during some examinations when I have to lie on the couch, the nurse helps me and while with me she stokes my hair, which makes me feel relax, and shows that she cares. When giving me injections, she asks me questions and as I attempt to answer her questions, my mind gets diverted from the pain of the injection, and I hardly feel the pain of injection being given'*. (Zaina)

*'The nurses helped with the injections and even though I was feeling sad and frustrated, the nurse helped me by consoling me'*. (Abeer).

*'During doctor's examination, the nurses helped me a lot because I was scared and really shy, I didn't know what to do, and they helped me by their presence to be examined without fear'*. (Nancy)

The above quotations illustrated how the participants perceived the presence of the nurse as relaxing and comforting during their treatment sessions.

## Discussion

### Psychological Impact of Infertility

The main indicators of emotional distress in the present study were anxiety, depression, reduced libido in both partners, and premature ejaculation and transient impotence among the men. In the qualitative interviews, in addition to the above more emotions were expressed including anger, feeling devastated, powerlessness, and sense of failure, frustration and overwhelming frustration. All the women interviewed confirmed that they were anxious, however, only 4 of their partners were said to be have been either sad, anxious or worried about their infertility diagnosis. The literature review also confirmed gender differences in the emotional reaction to infertility stating that women were more distressed by infertility whether they or their spouse caused the reproductive impairment [16,17]. This could be a result of social pressure from their relatives, in-laws, friends and even colleagues at work as confirmed during the qualitative interviews.

Most cultural orientations which teach that childbirth is a female's innate function, also contributes to this added pressure or stress in the infertile people. In some cultures, marriage is not considered successful until the couple bears a child and childbirth is a guarantee of the stability and perhaps insurance of the marriage.

Depression and reduced libido were more common among men than women. This was also associated with onset of smoking. Strained relationships and sexual difficulties appear to be central to male infertility-related stress. The degree of stress and desperation felt by infertile couples is emphasized by the fact that suicide among childless couples is approximately twice as frequent as couples with offspring [33]. However, the degree of desperation experienced may vary between clients depending on their situation: age, number of years married, whether primary or secondary infertility and result of investigations. In the qualitative interview, the younger participants less than 30 years with less than 3 years of infertility were not as devastated as the participants above 30 years with more than 5 years of primary infertility.

### Clients' perception of nurses' role during their treatment period

Most participants felt nurses who attended to them performed their basic nursing tasks like checking and recording vital signs and weight, giving injections and preparing them for examination or investigations. These were similar to the primary roles of direct patient care reported by 73% of infertility nurses reported in the literature review [22]. Some mentioned that nurses 'encouraged them', a term that had different connotations including: reassuring, communicating, counseling and educating them, similar roles were reported by nurses working in other fertility units [4].

### Implications for the Nursing Profession

Infertility is a difficult phenomenon to accept because of the complex psychosocial issues faced by infertile clients. The resultant emotional reactions can give rise to a vicious cycle of psychosexual problems, reduced pregnancy rates and obstetric outcomes. The response of the clients to the second major theme in the qualitative study revealed that the clients' perceived nurses presence during physical examination, investigations, embryo transfer and for the first hour after embryo transfer as comforting. They also reported that nurses communicating with them by clarifying doctor's instructions and providing health teaching on restrictions they must observed following embryo transfer were both educative and encouraging to them. There is therefore an implication for the nursing profession to develop fertility nursing as a sub-specialty with input from psychology to enable nurses in these settings to provide more structured psychological care. A survey of nurses who practice in infertility settings in the Western cultures revealed that 31% stated that they had been active in artificial reproductive nursing [21,22]. Unfortunately, this Kuwait study revealed that nurses working in fertility units are not trained to perform embryo transfer and counseling.

### The limitations of this study

In the quantitative survey, the couples were interviewed together in the clinic by the physician and co investigator. Interviewing them together may have restricted the response by each of them, however, this was necessary to ensure accuracy of their responses as it enabled them to recall and complement each others responses.

In the qualitative study, the perception of nurses' roles by 10 ART successful clients may not be representative of the perception of nurses' roles by all infertile clients, especially those still hoping to get pregnant. Having achieved pregnancy, these clients are most likely to report satisfactory care during treatment, contrary to what the unsuccessful client may feel about the treatment they are receiving. For the purpose of inter group comparison both successful and unsuccessful clients should have been included in the qualitative phase but the researchers were convinced that interviewing unsuccessful ART clients will be emotionally draining and too stressful.

Currently, there are relatively few studies addressing nurses' role in infertility management, the principal investigator believes that this study will form a baseline for future investigations.

## Conclusions

Nurses employed in fertility settings in Kuwait should be developed into specialist fertility nurses, through training and certification to enable them perform more expanded nursing roles like embryo transfer and counseling of distressed clients. Therapeutic communication is already included in all pre-registration nursing curricula, and counseling is part of post- basic nursing courses. Specialist infertility doctors in Kuwait should encourage their nursing personnel to engage in counseling their infertility clients if the need arises. Maternal health nursing courses in Colleges of Nursing should explore the possibility of collaborating with ART/IVF units in developing structured programs in fertility nursing as post-basic sub-specialty courses. The Infertility nursing certificate being offered in UK universities should be structured to attract international participation by offering summer holiday courses and E-learning modules to assist these nurses develop more knowledge and skills in infertility nursing. All the participants agreed that it would be useful if all the nurses working in fertility units were trained and certified fertility nurses.

### Recommendations

We recommend that nurses working in fertility clinics in Kuwait should have additional training in fertility nursing to enable them to organize and provide the following services to their clients:

• 24-hour bilingual (Arabic -English) telephone help line and counseling service.

• Distribution of bilingual information leaflet containing e-mail addresses, support websites or telephone numbers of international infertility support groups

• Establishment and co-ordination of local self-help/support group as a forum where infertile couples can meet and share their experiences, thus deriving strength from their different experiences.

• Establishment of educational programs to enlighten infertile couples about treatment options and answer their questions.

## Competing interests

The authors declare that they have no competing interests.

## Authors' contributions

FEO: participated in the design, literature review, data collection of semi structured interview of the 10 successful ART clients, statistical analysis and drafted the manuscript. AEO: conceived of the study, participated in the design, data collection using HSCL-25 and MFAS checklists, and performed the statistical analysis. Both authors read and approved the final manuscript

## Authors' information

FEO: RN, RM, BSc Nursing Education, M.Ed & PhD Health Education, Assistant Professor of Nursing and Chairperson of the Associate Degree of Nursing program at the College of Nursing, The Public Authority for Applied Education and Training, Kuwait.

AEO: MB. BS, MRCOG, FRCOG, Professor of Obstetrics and Gynecology at Kuwait University and Consultant at the Maternity Hospital, Kuwait.

## Appendix

### Interview Schedule

1. 'Why did you seek fertility treatment'?

2. 'How would you describe your feelings before and during your treatment for infertility'?

3. 'How did your spouse, family, in-laws and friends feel about your not getting pregnant'?

4. 'Please share with me your experiences with the nurses during your treatment period'?

5. 'What were your expectations of nurses' roles during your treatment?'

6. 'Which of the nurses' actions did you perceive as comforting'?

7. 'Which nursing actions did you perceive as caring or non-caring'?

8. 'Did the nurses acknowledge your emotional distress?

9. 'Which nursing actions made you feel they were distancing themselves from your problems'?

10. 'How did the nurses who attended to you show they understood your feelings'?

11. 'How did you assess nurses' communication with you'?

12. 'What aspects of their reaction made you feel they were listening or not listening to you'?

13. 'What aspects of their interactions made you feel they were reasoning with you during your communication with them'?

14. 'What do nurses do when they are in attendance during doctor's consultation with you'?

15. 'Did you feel the nurses in attendance were knowledgeable about your problems'?

16. 'How competent did you feel the nurses attending to you were'?

17. 'Do you have any suggestions to help improve the nurses working in infertility units'?

## Pre-publication history

The pre-publication history for this paper can be accessed here:

http://www.biomedcentral.com/1472-6955/9/5/prepub
